# Impact of Water Temperature on Heart Rate Variability during Bathing

**DOI:** 10.3390/life11050378

**Published:** 2021-04-22

**Authors:** Jianbo Xu, Wenxi Chen

**Affiliations:** Biomedical Information Engineering Laboratory, The University of Aizu, Aizu-Wakamatsu 965-8580, Japan; d8211103@u-aizu.ac.jp

**Keywords:** water temperature, bathing, ECG, heart rate variability, quantitative analysis, *t*-test

## Abstract

Background: Heart rate variability (HRV) is affected by many factors. This paper aims to explore the impact of water temperature (WT) on HRV during bathing. Methods: The bathtub WT was preset at three conditions: i.e., low WT (36–38 °C), medium WT (38–40 °C), and high WT (40–42 °C), respectively. Ten subjects participated in the data collection. Each subject collected five electrocardiogram (ECG) recordings at each preset bathtub WT condition. Each recording was 18 min long with a sampling rate of 200 Hz. In total, 150 ECG recordings and 150 WT recordings were collected. Twenty HRV features were calculated using 1-min ECG segments each time. The k-means clustering analysis method was used to analyze the rough trends based on the preset WT. Analyses of the significant differences were performed using the multivariate analysis of variance of *t*-tests, and the mean and standard deviation (SD) of each HRV feature based on the WT were calculated. Results: The statistics show that with increasing WT, 11 HRV features are significantly (*p* < 0.05) and monotonously reduced, four HRV features are significantly (*p* < 0.05) and monotonously rising, two HRV features are rising first and then reduced, two HRV features (fuzzy and approximate entropy) are almost unchanged, and vLF power is rising. Conclusion: The WT has an important impact on HRV during bathing. The findings in the present work reveal an important physiological factor that affects the dynamic changes of HRV and contribute to better quantitative analyses of HRV in future research works.

## 1. Introduction

Heart rate variability (HRV) is an important indicator of physical and mental health. The instantaneous HRV rhythm represents a dynamic balance between the sympathetic nervous system (SNS) and parasympathetic nervous system (PNS) branches of the autonomic nervous system (ANS) [1]. Therefore, the quantitative analysis of HRV is considered an effective method for the diagnosis of many cardiac diseases in clinical applications. However, many internal and external factors affect HRV. The internal factors include mental stress, gender, age, and disease, while the external factors include sleep, drugs, alcohol, smoking, and diet.

### 1.1. HRV and Stress

The SNS branch of the ANS was more activated during states of mental stress [2]; therefore, some literature evaluated mental stress using HRV analyses based on different stressors [3,4,5,6,7,8,9,10,11,12]. Some papers confirmed that the HR was significantly increasing during stress states [5,7,13,14,15,16,17,18,19,20], while some papers found that the mean R-R intervals (RRIs) [5,7,8,9,10,12,14,15,16,21] and the square root of the mean of the squares of the successive differences (RMSSD) between adjacent normal to normal intervals (NNs) [8,10,22,23,24,25,26,27,28,29] were significantly reduced during stress states. Kofman et al. discovered that the percentage of low frequency power in total power, pLF, was significantly higher while the percentage of high frequency power in total power, pHF, was significantly reduced during an examination stress state [4]. Melillo et al. found that the LF/HF ratio was significantly higher in the normal estimated glomerular filtration rate [30], while Hjortskov et al. proved that the LF/HF ratio was significantly higher during computer work stress states [3].

### 1.2. HRV and Gender and Age

The HRV dynamically changes with aging and gender. Ramaekers et al. and Schwartz et al. discovered that some HRV parameters decreased with aging, while Ramaekers et al. confirmed that the gender differences in the HRV parameters only exist in subjects younger than 40 years old [31,32]. Lochner et al. found that women’s HRV was significantly lower than men’s HRV [33]. Davy et al. observed that physically active women had higher levels of cardiac baroreflex sensitivity and HRV compared with sedentary women regardless of age [34]. Nagy et al. proved that gender differences determined HRV differences from birth, while boys’ HR baseline was significantly lower than that for girls [35]. Bonnemeier et al. noted that gender differences in HRV were significantly reduced with aging [36]. Yamasaki et al. discovered that LF was highly determined by aging and the pLF of men was significantly higher than that of women [37].

### 1.3. HRV and Disease

The HRV differs between healthy people and patients. Wilkowska et al. found that the HRV of depressed patients was significantly lower than that of nondepressed patients [38]. Lutfi and Sukkar showed that lower HRV was associated with higher BP values, putting subjects with such trends at a higher risk of developing hypertension [39]. T. Tombul et al. confirmed that lower HRV in multiple sclerosis patients than that in healthy [40]. D. Gurses et al. observed that some time domain parameters (mean RRIs, SDNN, RMSSD, and PNN50) were significantly lower in the thalassaemic patients then that of the healthy subjects [41]. M. Lan et al. found that the mean RRIs significantly increased, while LF% and LF/HF significantly decreased in the patients with allergic rhinitis in the sitting position [42]. DelRosso et al. investigated obstructive sleep apnea and found that it resulted in increased sympathetic activation during sleep in children [43].

### 1.4. HRV and Sleep

The sleep has an important impact on the HRV. Herzig et al. discovered that the HR was higher during REM sleep than during slow wave sleep (deep sleep) [44]. Padole and Ingale found that the HRV was distinguishable among the normal, sleeping, and meditation states [45]. Arslan et al. confirmed that the sleep deprivation resulted in a significant decreased in HF, TP, standard deviation (SD) of NN intervals (SDNN), and pNN50, with concomitant increased in the LF/HF ratio [46]. ÁR. Sűdy et al. confirmed that the HRV during sleep on workdays and free days was significantly different in young healthy men with social jetlag [47].

### 1.5. HRV and Other Factors

Many other factors also affect HRV. Hynynen et al. proved that the HRV of healthy men was significantly decreased, and the HR was significantly increased at night after marathon or moderate exercise sessions [48]. James et al. learned that the level of HRV significantly changed after severe intensity exercise [49]. Zuanetti et al. discovered that HRV significantly varied after patients took antiarrhythmic drugs [50]. Murgia et al. confirmed that HRV significantly increased after smoking cessation [51]. Young et al. learned that diet played an important role in the link between mood and HRV [52]. Latha et al. learned that classical music had a beneficial effect on HRV and reduced medical students’ stress levels [53]. Sollers et al. investigated the varying ambient temperature and found it had an important impact on the HRV [54]. Shin proved that ambient temperature induced a significant difference in pulse rate variability compared to HRV, and that the difference became greater at a higher ambient temperature [55].

Some previous studies explored the impact of water temperature (WT) on HRV. Mourot et al. and HC. Choo et al. found that immersion in different WT had an important impact on the HRV [56,57]. Y. Kataoka et al. studied the impact of WT on HRV during bathing, but only 38 °C and 41 °C were included, and a few HRV measures were evaluated [58]. F Edelhäuser et al. investigated the effects of whole-body immersion on HRV at three different WTs (33 °C, 36 °C, and 39 °C) [59].

The main purpose of this paper aims to explore the impact of different WTs on HRV during bathing. The experiment was carried out based on the most commonly used WTs in the daily family life, twenty HRV features (included time domain, frequency domain, and non-linear domain) were calculate.

## 2. Method

### 2.1. ECG Collection System

The electrocardiogram (ECG) collection system in this study includes four rectangular stainless steel noncontact electrodes, all of them placed on the bathtub wall. When the subject is in the bathtub during bathing, the four noncontact electrodes are near the right foot, right arm, left foot, and left arm, respectively.

**Figure 1:**
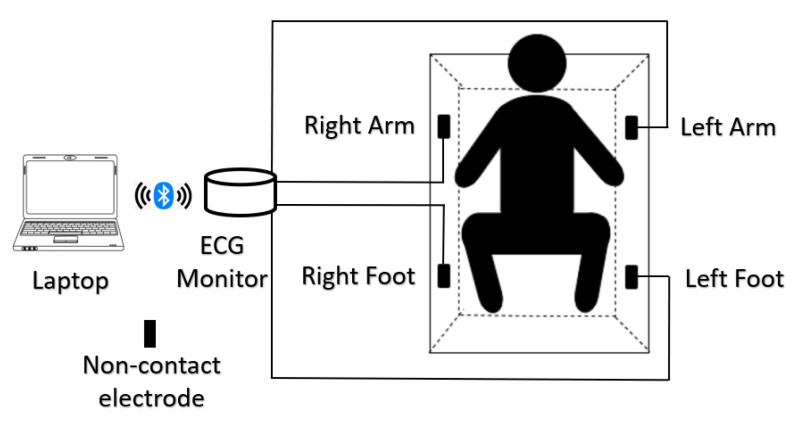
ECG collection system.

The electricity on the skin surface, which is produced by the electrical activity of the heart, arrives in the four noncontact electrodes through the water and three-lead ECG are recorded. The lead I ECG is the potential difference between the left arm (positive) and right arm (negative), the lead II ECG is the potential difference between the left foot (positive) and right arm (negative), and the lead III ECG is the potential difference between the left foot (positive) and left arm (negative). Four shielded wires are, respectively, welded onto the four noncontact electrodes. The three-lead ECG arrives in the ECG collection monitor (Open Brain Computer Interface Biosensing Ganglion Board-OpenBCI Ganglion; OpenBCI, USA) through the shielded wires, and the ECG monitor and the laptop (a MacBook Pro) are connected using a standard Bluetooth 4.n, and all the collected ECG recordings are stored on the laptop. The designed ECG collection system in this study is shown in Figure 1 [60].

### 2.2. Subjects and ECG Recordings

The ECG recording procedures were approved by the Public University Corporation, the University of Aizu Research Ethics Committee. Written informed consent was obtained from each participant before the experiment.

Ten subjects (seven men and three women) aged 23 to 40 years old (mean ± SD: 28.5 ± 4.78 years) who were students from the University of Aizu participated in the data collection. The BP, body temperature, and body weight were recorded before and after the ECG collection, and the temperature profile for WT change and room temperature were recorded every second during the ECG collection using a temperature monitor (TR-71wb/nw; T&D Corporation, 817-1, Shimadachi, Matsumoto, Nagano, Japan, 390-0852).

The preset bathtub WT includes three conditions: low WT (36–38 °C), medium WT (38–40 °C), and high WT (40–42 °C), respectively. Each subject collected 5 ECG recordings at each preset bathtub WT condition and each recording was 18 min long with a sampling rate of 200 Hz. In total, 150 ECG recordings and 150 temperature recordings were collected during bathing.

### 2.3. ECG Processing

**Figure 2:**
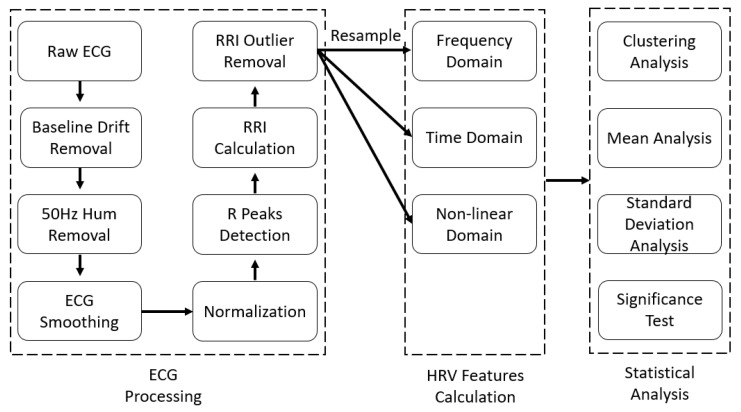
Flowchart for the ECG processing, HRV feature calculation, and statistical analysis.

The flowchart for the ECG processing, HRV feature calculation, and statistical analysis is shown in Figure 2.

All data processing and analyses were performed using MATLAB (R2019a). Baseline wandering is obvious in the raw ECG signal due to motion artifacts and respiration from the subjects; therefore, the wandering baseline was removed using the one-dimensional (1-D) wavelet decomposition and reconstruction methods. The Daubechies wavelet at level 10 was used to decompose the raw ECG signal and the baseline wandering approximation coefficient was subtracted from the raw ECG signal after reconstructing at level 8.

Obvious hum noise was also observed in the raw ECG signal; therefore, we performed a spectrum analysis on the raw ECG signal. The spectrum analysis results show that the main frequency component of the hum noise was 50 Hz, which is mainly produced by the electromagnetic interference between the power supply network and equipment [61]. A second-order infinite impulse response digital notch filter was used to remove the 50 Hz hum noise. The numerator and denominator coefficients of the digital notch filter with the notch located at ω and the bandwidth at 0.0071 at the −3 dB level were calculated and the ω must meet the condition of 0.0 < ω < 1.0. The difference equation of the digital notch filter is shown in Equation (1).

(1) $$y\left[n\right]=\sum_{i=0}^{N}b_{i}x\left[n-i\right]-\sum_{i=1}^{M}a_{i}y\left[n-i\right],\;\;n\geq 0$$

where *x*[*n*] is the filter input, *y*[*n*] is the filter output, and *a${}_{i}$* and *b${}_{i}$* are the numerator and denominator coefficients, respectively, of the digital notch filter.

Next, the 5-point moving average method was used to smoothen the ECG signal. The mathematical formula for the moving average is shown in Equation (2):

(2) $$y\left[n\right]=\frac{1}{M}\sum_{j=0}^{M-1}x\left[n-j\right]$$

where *x*[*n*] is the input signal, *y*[*n*] is the output signal, and *M* is 5.

Then the ECG was normalized into the range of 0 to 1 using the “mapminmax” function, the R peaks were detected using the “findpeaks” function, the RRI were calculated, and the RRI outliers removed using the 1D 11th order median filter because of its outstanding capability in suppressing the isolated outlier noise without blurring sharp changes in the original signal.

The mathematical formula of the 1D 11th order median filter is shown in Equation (3):

(3) y[i]=median{x[i],i∈w}

where *x*[*i*] is the input signal, *y*[*i*] is the output signal, and *w* is the moving window length.

**Figure 3:**
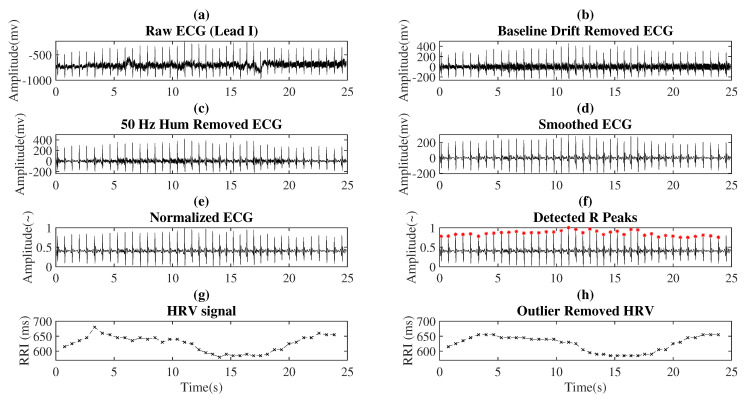
Output of the ECG processing steps.

The results for each ECG processing step are shown in Figure 3.

### 2.4. HRV Analysis

HRV analysis methods include linear and nonlinear domain analysis methods, where the linear domain includes time and frequency domain methods. The HRV features in the time domain include HR, mean RRI, SDNN, RMSSD between adjacent NNs, SD of the successive differences between adjacent NNs (SDSD), and area under RRI (AURRI). The HRV features in the frequency domain include very LF (VLF) power (0.003–0.04 Hz), LF power (0.04–0.15 Hz), HF power (0.15–0.4 Hz), total power (0–0.4 Hz), pLF, pHF, and the LF/HF ratio. The HRV features in the nonlinear domain include the correlation dimension (D${}_{2}$), the SD of the Poincare plot perpendicular to the line of identity (SD1), the SD of the Poincare plot along to the line of identity (SD2), the SD1/SD2 ratio, and the sample (SE), fuzzy (FE), and approximate entropies (AE).

Before the frequency features are calculated, a RRI resample is necessary. According to Nyquist’s sampling theorem, the sample rate must be more than two times the highest frequency in the original signal. The highest frequency of the HRV is 0.4 Hz; therefore, the new resampling rate for RRI was set at 2 Hz. Then, a discrete Fourier transform (DFT) was used to calculate the power spectral density (PSD) of the resampled RRI for a N points sequence. Its DFT is shown in Equation (4):

(4) $$X\left[k\right]=\sum_{n=0}^{N-1}x\left[n\right]e^{-i\frac{2\pi }{N}nk}.$$

where *k* = 0, 1, 2, ... , N−1, and $i^{2}$ = −1.

### 2.5. Statistical Analysis of the HRV Features

Each ECG recording was 18 min in length and segmented into 18 equal parts. A 1-min ECG was used to calculate the HRV features each time. Based on the bathtub WT, the mean and the SD of each HRV feature were calculated, and the *t*-test was used to test for significance. The summary statistic results of each HRV feature are visualized in the clustering results and box plot.

## 3. Results

**Figure 4:**
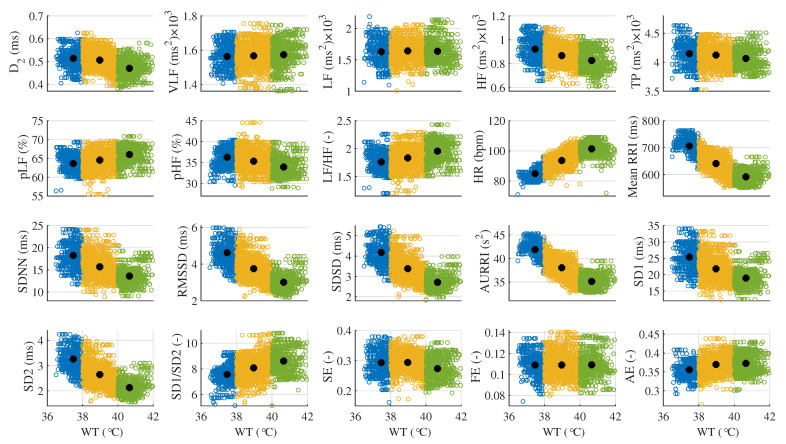
Feature trends for 20 HRV features in three analysis domains under three WT conditions.

The variations of the HRV features based on different WTs are shown in Figure 4. The smaller dots with blue, yellow, and green colors represent the HRV features calculated based on each preset WT, while the bigger black dots are the average values of the HRV features based on each preset WT calculated by the K-means clustering analysis method. For the areas of the dots of HR, the blue area is smallest, the yellow area is biggest, and the green area is medium sized. The low WT condition is very close to the average temperature (about 36.5 °C) of the normal human body, therefore, the WT stimulation was not strong to the subjects, with a small variation in HR and the SD of HR was 3.38. The higher WT has a stronger stimulation to the subjects during bathing. Although the instantaneous HR was very fast at this WT condition, the HRV was not the biggest with a SD of HR 4.17. The stimulation for the medium WT condition was bigger than the stimulation for the low WT condition, but was smaller than the high WT condition. The HRV is obvious with a SD of HR 4.65; therefore, the area of the yellow dots was the biggest.

Figure 4 shows that the controlled condition of WT was not serious or uniform. In fact, the low WT was not strictly and evenly distributed in the range of (36–38 °C) and was far below the ambient temperature during bathing. The WT of 42 °C was far beyond the ambient temperature during bathing; thus, the WT decreased quickly during the data collection and the WT data at about 42 °C were not as concentrated. It is obvious that the D${}_{2}$, HF power, total power, pHF, mean RRI, SDNN, RMSSD, SDSD, AURRI, SD1, and SD2 were monotonously reduced with increasing WT, and the pLF, LF/HF, HR, and SD1/SD2 were monotonously rising with increasing WT. A significant difference (*p* < 0.05) was found among the above HRV features.

**Figure 5:**
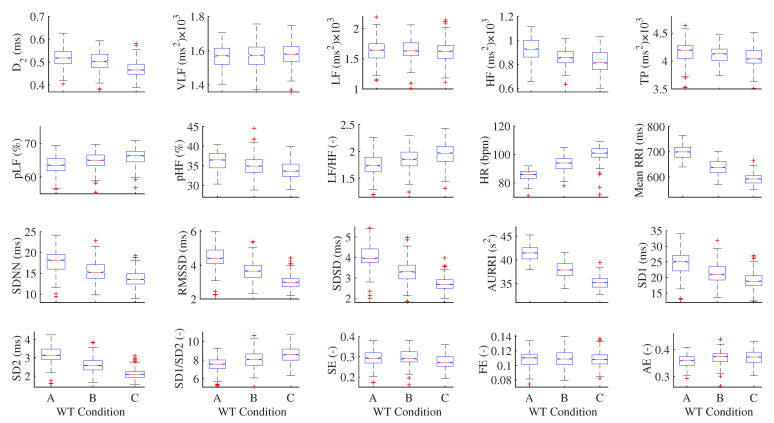
Analysis of significant differences for 20 HRV features in three analysis domains under three WT conditions: A = (36–38) °C, B = (38–40) °C, and C = (40–42) °C.

The results of significant difference analyses for the 20 HRV features in three analysis domains under three WT conditions are visualized in Figure 5. There are some outliers for each HRV feature. For the HR, the higher of the WT, the more outliers because the subjects experienced stronger stimulation from the higher WT and it is more difficult to adapt the WT environment during bathing. The changes in the mean of VLF, LF, SE, FE, and AE were not obvious, and significant differences were not found in these five HRV features.

The details of the statistic results of the 20 HRV features in the time, frequency, and nonlinear domains are shown in Table 1, where the mean values and SD are listed, and the pairwise statistically significant differences between each WT condition are calculated. The significant difference analysis was performed via the multivariate analysis using the *t*-test variance method, where *p1* represents the significant difference between low and medium WT conditions, *p2* represents the significant difference between medium and high WT conditions, and *p3* represents the significant difference between low and high WT conditions. With the increasing WT, the SD of the HR, mean RRI, AURRI, pLF, pHF, LF/HF ratio, and SD1/SD2 are first rising and then reduced, and the SD of LF, HF, TP are first reduced and then rising.

## 4. Discussion

As a noninvasive, rapid, and accurate tool in the evaluation of the cardiac autonomic balance modulation activity, heart rate variety (HRV) has been a hot research topic in recent years. This study explored the impact of different water temperature (WT) on HRV during bathing. With the rises of WT, the HR in medium and high WT increased by 6.53% and 15.78%, respectively, compared with the low WT, which reflects a decreased vagal modulation. The significantly and monotonously reduced SDNN with increasing WT shows a significantly reduced whole HRV fluctuation, which is highly consistent with the significantly and monotonously reduced total spectral power (0–0.4 Hz). The LF power (0.04–0.15 Hz) in the PSD reflects both SNS and PNS activities, while the HF power (0.15–0.4 Hz) in the PSD reflects the PNS activity, and the LF/HF ratio represents the balance between the SNS and PNS activities. With the increasing WT, the LF and HF are significantly and monotonously reduced, which reflects that SNS and PNS activities are enhanced significantly. The increased LF/HF ratio shows that the ratio of the cardiac sympathetic to parasympathetic tones (i.e., the sympathovagal balance) was enhanced significantly, which shows that the stimulation of high WT on the subject was also enhanced significantly. The stimulation on the subject under high WT increased by 6.43% and 5.20% over the low and medium WT, as shown in Table 1. Furthermore, the HRV feature of AURRI was newly defined in this paper and its unit is $s^{2}$. The AURRI reflects the fluctuation of HRV signal over time: i.e., with the increasing WT, the mean RRI and AURRI are reduced. In lower WT condition, the parasympathetic activity is dominant. With the WT increasing, our findings show decreased HRV complexity, which induce obvious shift of ANS balance towards the sympathetic activation associated with vagal withdrawal. Therefore, the higher WT can induce a stronger response of physiological allostatic regulatory, which is often accompanied by an enhanced cardiac sympathoexcitation associated with a vagal withdrawal. From the healthcare perspective, to reduce the sudden onset possibility of cardiac and cardiovascular complications or diseases during bathing, it is more dangerous to choose a higher WT condition for the patients.

The same WTs which belonging to different WT change processes induce different impacts on HRV. For example, if the WT drops from 40 °C to 38 °C during the data collection process, the subject will feel very uncomfortable in the first minute and need a longer time to adapt to the WT environment. However, if the WT increases from 38 °C to 40 °C during the data collection process, the subject will adapt to the WT environment easily. Even if the WT reaches 40 °C, the subjects will not feel very uncomfortable because they have adapted to this WT environment. The WT of 40 °C appeared during two different processes, but had very different instantaneous effects on the HRV and their physiological meanings were also different in these processes. Therefore, some outliers appear in the box plot as shown in Figure 5.

**Figure 6:**
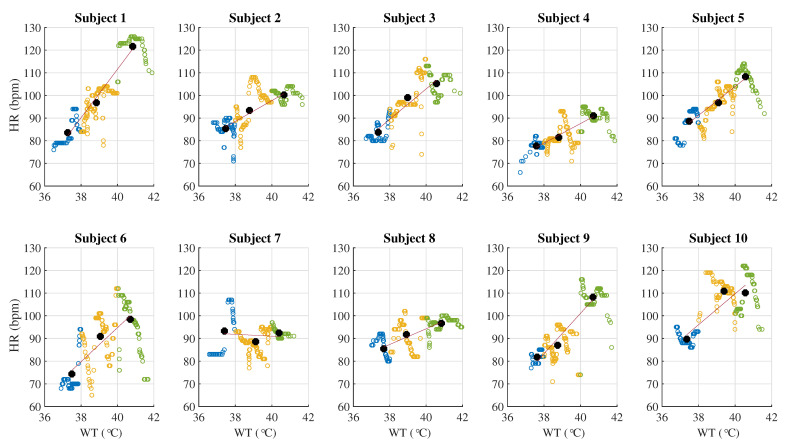
The variety of HR and its fitted curve at one degree for each subject under three WT conditions.

According to experiment records, the difference from other subjects was that Subjects 4, 7, and 8 did not feel very uncomfortable even in the higher WT (40 °C–42 °C).The slopes of the variety of HR are smaller than that of the other seven subjects, as shown in Table 2. Specifically, Subject 7 preferred the higher WT and the change in their HR was not as obvious as in the other subjects, as shown in Figure 6. The questionnaire showed that Subject 7 often participated regular physical activities. Regular exercise could make the sympathetic-adrenal system more easily excited, thus enhancing cardiovascular, hormonal and metabolic responses, further affecting body temperature regulation, water-electrolyte interface, muscle contraction performance, etc., thus ensuring blood perfusion, oxygen, and nutrient supply and elimination of metabolites in organs and tissues throughout the body. There was evidence that exercise could reduce the sympatho-excitation and sympathetic outflow, and the baroreflex-mediated was also suppressed. Therefore, compared with other subjects, Subject 7 demonstrated higher HRV, and their reaction to higher WT indicated a great adaptability of the ANS.

Subjects 1, 6, and 9 were very sensitive to changes in WT and especially could not tolerate the high bathtub WT (40–42 °C). They felt more comfortable during 4th–11th min on the data collection process. The first 3 min are the adaptation phase. During this stage, their foreheads quickly began to sweat a lot. For the other seven subjects, their adaptation phases were the first 1 min and they began to sweat more after the first 10 min in the same high WT environment. The body weights of these three subjects were reduced more after data collection than in the other seven subjects. From this finding, we speculate that people who are more sensitive to temperature changes are less able to withstand water and WT pressures, and they are more likely to suffer from higher mental and physical stress during the higher WT condition.

Subjects 2, 3, 5, and 10 felt a little uncomfortable, but could tolerate the high WT (40–42 °C). All ten subjects could quickly adapt to the low WT (36–38 °C) and they felt more relaxed and comfortable in the medium WT (38–40 °C). Except for three subjects who were very sensitive to the WT changes, the other seven subjects did not feel uncomfortable.

Although some discoveries were revealed in this paper, there are also some limitations. First, the set-up of the experiment is stressful itself, and therefore may create an additional bias. Although informed consent was obtained and the data collection process was told in detail to each subject, as well as let each subject had a five-minute rest before the data collection, some subjects were still a little nervous at the beginning of data collection. Furthermore, the sensitivity to external stimuli of each subject was different, the water pressure on the chest and thermal stimulus on hemodynamics also induced different stress to each subject, which would induce some additional biases to the results. Therefore, the mental stress factor should be also taken into consideration at the same time to evaluate the impact of WT on HRV. Second, the number of subjects is too small and the subjects should include older people and children, in addition to healthy and unhealthy individuals and different ethnicities. Third, the change ranges for WT during the data collection were too big. Due to poor insulation measures, the WT was relatively divergent during the data collection process. Thus, the HRV analysis should be performed based on several smaller ranges of WT. Fourth, the data collection environment was inconsistent for all the subjects. For example, when the WT is between 40 °C to 42 °C, some subjects could endure the high WT environment, while other subjects felt too uncomfortable to endure the high WT environment. Therefore, to be safe, we must give these subjects a fan to blow a refreshing cool breeze on themselves in this situation. Fifth, during the data-processing stage, the median filter was used three times to remove the outliers of the RRI signal. The skin surface electricity is very weak, in the millivolts. The gentle movement of the four limbs will induce relatively large fluctuations in the ECG amplitude. Therefore, the raw ECG signal includes some noise and there are some outliers in the R peaks detection and RRI signal results. If the median filter is only used once to remove the RRI outliers, then either only the outliers with big amplitude can be removed or there is a gross distortion in the RRI signal after the outliers are removed. Therefore, the median filter was used to remove the outliers with big, median, and small amplitudes, respectively.

## 5. Conclusions and Future Work

This paper explores the impact of different WTs on HRV during bathing. With the WTs increasing, some HRV features are significantly and monotonously reduced or rising, which induces the change of dynamic balance between the SNS and PNS branches of the ANS. The findings in the present study provide important reference significance in many practical aspects which need to evaluate the amount of disturbance of homeostasis induced by WTs. For example, we can affect the HRV by changing the WTs to set an optimal environment during bathing. Only when the SNS and PNS activities are controlled at a certain range can the people feel relaxed and comfortable.

In future research works, we will further explore the HRV levels of healthy subjects and patients, especially the patients with cardiac diseases (such as arrhythmia, myocardial ischemia, and coronary heart disease), and then design an automated and accurate WTs control system to affect the HRV by changing the WTs so that the HRV level is indirectly controlled in a safe and comfortable range based on individual health condition, which would appropriately reduce the possibility of sudden onset of cardiac disease during bathing. Moreover, in order to achieve the purpose of lifelong healthcare, we will also explore how to apply the cutting-edge blockchain technology in the long-term tracking of ECG data during bathing for the big data collection and analysis [62,63]. Another particularly crucial research topic is the physiological signal encryption and secure transmission related to the privacy protection, some emerging technologies provide a valuable reference [64,65].

## Figures and Tables

**Table 1:** The statistic results of the HRV features based on different bathtub WT.

HRV Features		Features	(36 38] °C		(38 40] °C		(40 42] °C		*p* Value		
		Trend	Mean	SD	Mean	SD	Mean	SD	*p1*	*p2*	*p3*
Time	HR (bpm)	↑↑	85.55	3.38	94.07	4.65	101.30	4.17	0	0	0
Domain	Mean RRI (ms)	↓↓	699.93	27.53	637.04	30.67	591.15	22.42	0	0	0
	SDNN (ms)	↓↓	17.99	2.94	15.48	2.48	13.70	1.87	0	0	0
	RMSSD (ms)	↓↓	4.54	0.74	3.69	0.57	3.03	0.42	0	0	0
	SDSD (ms)	↓↓	4.11	0.65	3.32	0.50	2.72	0.35	0	0	0
	AURRI (s${}^{2}$)	↓↓	41.51	1.62	37.82	1.80	35.12	1.32	0	0	0
Frequency	VLF Power (ms${}^{2}$)	↑	1561.77	78.82	1567.90	87.13	1573.03	80.22	0.07	0.14	0
Domain	LF Power (ms${}^{2}$)	∧	1631.05	182.50	1646.35	159.13	1633.92	175.79	0.04	0.11	0.74
	HF Power (ms${}^{2}$)	↓↓	922.03	97.45	859.79	68.12	826.23	86.79	0	0	0
	Total Power (ms${}^{2}$)	↓↓	4157.74	199.76	4114.04	158.49	4065.92	159.75	0	0	0
	pLF (%)	↑↑	63.53	2.37	64.75	2.58	65.91	2.29	0	0	0
	pHF (%)	↓↓	36.36	2.37	35.13	2.45	33.97	2.20	0	0	0
	LF/HF (-)	↑↑	1.75	0.18	1.85	0.20	1.95	0.19	0	0	0
Non-linear	D${}_{2}$ (ms)	↓↓	0.52	0.04	0.51	0.04	0.47	0.04	0	0	0
Domain	SD1 (ms)	↓↓	24.94	4.10	21.51	3.48	19.11	2.62	0	0	0
	SD2 (ms)	↓↓	3.21	0.53	2.61	0.40	2.14	0.30	0	0	0
	SD1/SD2 (-)	↑↑	7.63	0.82	8.03	0.86	8.65	0.85	0	0	0
	SE (-)	∧	0.29	0.04	0.30	0.03	0.27	0.03	0.15	0	0
	FE (-)	∼	0.11	0.01	0.11	0.01	0.11	0.01	0.21	0.04	0.43
	AE (-)	∼	0.36	0.02	0.37	0.02	0.37	0.02	0	0.14	0

⇊ (⇈): Significantly reduced (increase) with increasing WT *p* < 0.05; ↓ (↑): Reduced (increase) with increasing WT *p* > 0.05; ∧: Increased first and then reduced with increasing WT; ∼: Unobvious change; *p1* = between (36–38) °C and (38–40) °C; *p2* = between (38–40) °C and (40–42) °C; *p3* = between (36–38) °C and (40–42) °C. (36–38) °C: 36 < WT≤38 °C; (38–40) °C: 38 < WT≤40 °C; (40–42) °C: 40 < WT≤42 °C.

**Table 2:** The slope of the variety of HR with increasing WT.

Subject	1	2	3	4	5	6	7	8	9	10
Slope	10.71	4.59	6.72	4.34	6.36	7.48	−0.41	3.46	8.91	6.83

## Data Availability

Data are available from the biomedical information engineering laboratory (contact via e-mail: wenxi@u-aizu.ac.jp) for researchers who meet the criteria for access to confidential data.
